# Effect of Ionic Surfactants on Kinetics and Mechanism of the Bi(III) Ion Electroreduction in the Mixed Aqueous–Organic Solutions of Supporting Electrolytes

**DOI:** 10.3390/molecules29214986

**Published:** 2024-10-22

**Authors:** Alicja Pawlak, Agnieszka Nosal-Wiercińska

**Affiliations:** Department of Analytical Chemistry, Institute of Chemical Sciences, Faculty of Chemistry, Maria Curie-Sklodowska University, Maria Curie-Sklodowska Sq. 3, 20-031 Lublin, Poland; alicja.pawlak@mail.umcs.pl

**Keywords:** electrochemistry, R-AgLAFE electrode, surfactants, bismuth(III), methanol, active complexes, kinetics parameters

## Abstract

This work presents the results of a study on the effect of ionic surfactants: cationic hexadecyltriammonium bromide (CTAB) and anionic sodium salt of sulfonic acid (1OSASS) on the Bi(III) electroreduction process in mixed aqueous–organic supporting electrolyte solutions containing methanol. This study showed that the composition of the supporting electrolyte solution, particularly the methanol and surfactant concentrations, significantly affects the mechanism and rate of the Bi(III) ion electroreduction. Analysis of the influence of the indicated factors on the mechanisms and kinetics of metal ion electroreduction can contribute not only to the optimization of industrial electrochemical processes but also to the development of innovative technological solutions, such as advanced electrochemical materials and novel sensors. In these experiments, an innovative electrode made of cyclic renewable liquid silver amalgam (R-AgLAFE) was used as a working electrode, which stands out among classic mercury electrodes (HMDE type) due to the significant reduction in mercury consumption while maintaining similar performance.

## 1. Introduction

The rate of an electrode reaction is determined by a variety of factors, such as the composition of the supporting electrolyte (SE) as well as the presence of other substances that affect the properties of both the electrode and the solution. Organic solvents alter the physicochemical properties of mixed aqueous–organic solutions, such as polarity, viscosity, and ion solvation capacity, which can lead, for example, to modification of the mechanisms of adsorption processes in the electric double layer (EDL) [1,2].

In practice, control of electrode reaction rates is crucial for various applications. Choosing the right supporting electrolyte composition can significantly affect parameters such as performance, capacity, and lifetime of, for example, lithium-ion batteries. In the context of pollutant removal efficiency in electrochemical water purification, the kinetics of electrode reactions also play a key role. It is mainly governed by both the electrolyte composition and the presence of electrode surface modifiers. In addition, in industrial electrolysis processes for the production of reagents (such as gases, metals, acids, or bases), control of the electrical properties of the double layer allows optimization of the energy efficiency and selectivity of chemical reactions. Appropriate control of reaction parameters can therefore be extremely important not only for the development of new electrochemical sensors but also for the optimization of existing industrial and technological processes.

Studies on the metal ion electroreduction in the presence of organic substances contribute to a better understanding of the influence of these compounds on the kinetics and mechanism of electrode processes, which follows the so-called “cap-pair” rule, formulated in 1978 by Sykut’s research team [3]. This rule defines criteria for the catalytic activity of a given organic substance, which should contain sulfur or nitrogen atoms in its structure and have free electron pairs capable of forming active complexes with depolarizer ions in the electrode layer. In addition, in the reduction potential range of the depolarizer, the substance should be labilely bound to the electrode surface. It has been shown that in aqueous solutions of supporting electrolytes, appropriately selected organic substances can act as both catalysts and inhibitors of the electrode processes [4,5,6,7].

Current research trends largely focus on the use of special electrodes, albeit carbon paste-based electrodes (CPEs) [8,9], which are often modified with surfactants that affect electrode processes. However, the aforementioned “cap-pair” principle does not apply to this type of sensor. This limitation underscores the need to use alternative electrode materials in research. In this work, we used a cyclic renewable liquid silver amalgam electrode (R-AgLAFE), which allowed us to study the kinetics and mechanism of the Bi(III) iona electroreduction reaction in the presence of the surfactants, i.e., cationic hexadecyltrimethylammonium bromide (CTAB) and anionic sulfonic acid sodium salt (1OSASS), in systems containing methanol as an organic component of mixed aqueous–organic supporting electrolytes.

The research aimed to understand the effects of these modifications on the electrochemical properties of the systems and the kinetics of the reactions taking place, which opens up new possibilities in the creation of, for example, advanced materials for electrochemical applications used both in industry and medicine—such as modern sensors, functional materials, or in metal deposition procedures.

## 2. Results and Discussion

### 2.1. Polarographic, Voltammetric, and Impedance Measurements

Studies of acceleration of electrode processes in mixed aqueous–organic electrolytes in terms of the “cap-pair” rule concerned two electron depolarisers, mainly Zn(II) ions [5,10]. According to this rule, organic substances can both accelerate and slow down processes, depending on the appropriate choice of substances. It has been shown that the kinetics of the electrode process are decisively influenced by the rate of transfer of the first electron. In addition, it has been proven that the formation reaction of the active complex plays a key role in the acceleration process [11,12].

The “cap-pair rule” can also help determine the mechanism and kinetics of the electroreduction of three electron ions in mixed aqueous–organic electrolyte solutions.

**Figure 1:**
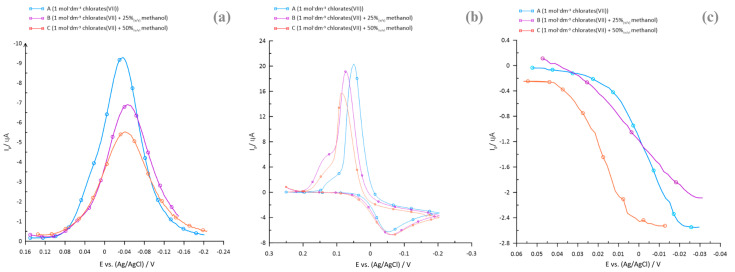
(**a**) SWV peaks of the 1 × 10^−3^ mol·dm^−3^ Bi(III) ion electroreduction in aqueous–organic solutions of supporting electrolyte. (**b**) Voltammetric CV curves of the 1 × 10^−3^ mol·dm^−3^ Bi(III) ion electroreduction of in aqueous–organic supporting electrolyte solutions. (**c**) DC polarographic waveforms of the 1 × 10^−3^ mol·dm^−3^ Bi(III) ion electroreduction in aqueous–organic solutions of the supporting electrolyte.

**Figure 2:**
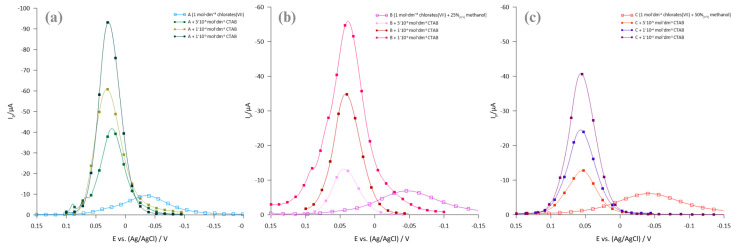
(**a**–**c**). The SWV peaks of the 1 × 10^−3^ mol·dm^−3^ Bi(III) ion electroreduction in SEs (A–C) solutions in the presence of selected concentrations of CTAB are indicated in the legend.

**Figure 3:**
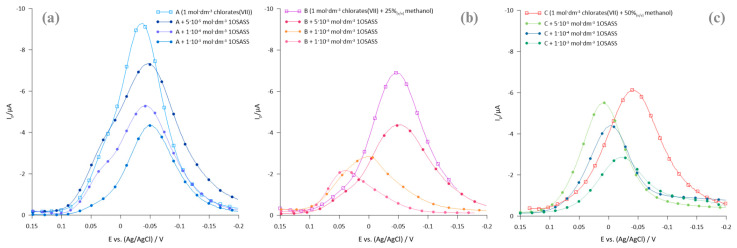
(**a**–**c**) SWV peaks of the 1 × 10^−3^ mol·dm^−3^ Bi(III) ion electroreduction in SEs (A–C) solutions in the presence of different concentrations of 1OSASS indicated in the legend.

In Figure 1, Figure 2 and Figure 3, indicate the correlations obtained from polarographic and voltammetric measurements, which allow qualitative evaluation of the effect of methanol present in the base electrolyte on the rate of the electrode process. Both the presence and the increase in the amount of methanol in the solution of the supporting electrolyte cause a decrease in the reversibility of the Bi(III) ion electrodeposition process. This is evidenced by a decrease in the SWV peak currents (Figure 1a), an increase in the anodic and cathodic peak potential difference (ΔE) in the CV curves (Figure 1b), and a decrease in the slope of the DC polarographic waveform (Figure 1c). The observed potential shifts toward more negative values, coupled with a concomitant decrease in the intensities of the peak currents on the square wave voltammetry curves (Figure 1a), along with an increase in the concentration of methanol in the SE solutions, may indicate that the electroreduction process of Bi(III) ions was inhibited by the adsorption of methanol on the electrode surface. The observed effects also suggest a significant influence of electrostatic interactions in the SE chlorate(VII)–methanol system, which led to a decrease in charge transfer efficiency.

The presence of CTAB in the tested solutions changes the kinetics of the Bi(III) ion electroreduction process in the direction of acceleration, which is confirmed by the increases in SWV peak currents and the decreasing width of the peaks in the middle of their height (Figure 2a–c). A shift in SWV peaks after the introduction of the cationic surfactant (CTAB) is also observed, which may be due to strong electrostatic interactions between positively charged CTAB ions/micelles and depolarizer ions present in the solution, as well as interactions with the R-AgLAFE electrode surface.

The addition of 1OSASS causes a decrease in the height of the SWV peak current (Figure 3a–c) in all SE solutions studied. The dynamics of the Bi(III) ion electroreduction in mixed electrolyte solutions (chlorate(VII)—methanol), due to the presence of CTAB and 1OSASS, is clearly changed. With increasing methanol content in EP solutions, peak currents decrease and become less well-defined, indicating a decrease in the reversibility of the electrode process [13] (Figure 3a–c). The lack of peak shift in the presence of anionic surfactant suggests that 1OSASS does not induce similar electrostatic interactions as CTAB, which may be due to the difference in ionicity of the two surfactants. Anionic surfactants can induce repulsive forces at the electrode/solution interface, which is not conducive to the formation of active complexes that facilitate electron transfer in the electrode process.

The influence of CTAB and 1OSASS and variations in the $MeOH-ClO_{4}^{-}$ concentration ratio (in a system without the presence of surfactants) on the reversibility of the Bi(III) ion electroreduction process was also confirmed by data obtained from cyclic voltammetry measurements. As the amount of methanol in the SE solution increases, an extension of the distance between the anodic and cathodic peaks is observed (ΔE_A_ = 0.073; ΔE_B_ = 0.090; ΔE_C_ = 0.098), which translates into a slowdown in the process rate.

In the presence of CTAB, ΔE values are decreasing (ΔE_B_ = 0.090; ΔE_B1_ = 0.078; ΔE_B2_ = 0.045; ΔE_B3_ = 0.032), while in the presence of 1OSASS, they register an increase (ΔE_B_ = 0.090; ΔE_B1_ = 0.094; ΔE_B2_ = 0.104; ΔE_B3_ = 0.106).

Previous studies have pointed out the complex mechanism of the Bi(III) ion electroreduction process on a mercury electrode [14] and suggested a chemical reaction for the formation of active complexes that are involved in electron transition [15]. They also suggest a multi-step electrode mechanism in the examined systems. It should be noted that the organic component of the mixed supporting electrolyte can, in a certain range of electrode potentials, adsorb [16], modifying the phase interface or changing the nature of the initial ion in the electrode reaction.

**Figure 4:**
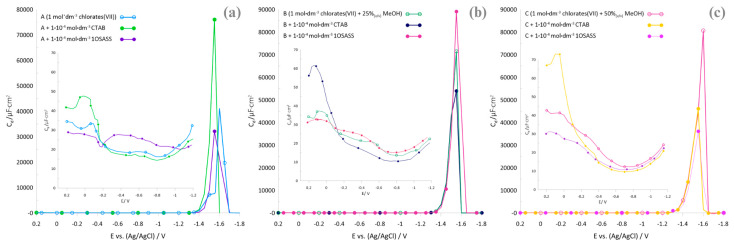
(**a**–**c**). Extrapolated to zero-frequency differential capacitance curves, the electrical double layer of the R-AgLAFE/chlorate(VII) and R-AgLAFE/chlorate(VII)–methanol in the presence of surfactants.

The presence of the surfactant CTAB and 1OSASS (with concentrations of 1 × 10^−4^ mol·dm^−3^) changes the capacitance curves obtained for the supporting electrolyte alone, indicating the adsorption of surfactants on the electrode [17] (Figure 4a–c). 

**Figure 5:**
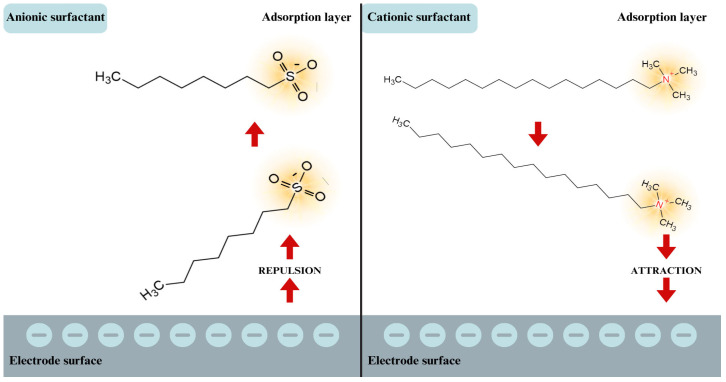
Electrostatic interactions between the negatively charged surface of the R-AgLAFE electrode and the hydroxylated ions of the CTAB and 1OSASS surfactants.

At extremely positive potentials, an adsorption peak appears, but only in the case of CTAB, while in the presence of 1OSASS, only its outline is observed. In the case of a highly negative electrode potential (about −1.5 V), desorption peaks are observed. The different picture of the changes in the C_d_ = f (E) curves in the studied systems should be associated with the structure of the surfactants in terms of their ionality (CTAB—cationic; 1OSASS—anionic), which probably is related to a different arrangement of them on the surface of the R-AgLAFE electrode (Figure 5).

Literature data strongly suggest the physical adsorption of these surfactants on the mercury electrode [18,19]. It should be noted that the image of the capacitance curves also varies with a change in the composition of the base electrolyte, which indicates the influence of the molecules of the organic component (in this case, methanol) on the composition of the surface layer interface [4,20]. The adsorption–desorption peaks modify their position and shape, which may indicate changes in the interactions (especially electrostatic) between the adsorbed molecules of a given surfactant and methanol and the formation of a mixed adsorption layer [21].

**Figure 6:**
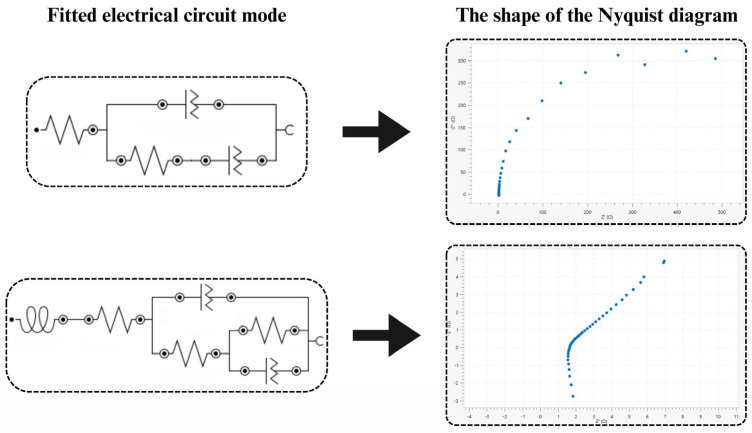
Electrical replacement systems used to determine the R_a_ activation resistance.

The catalytic influence of the cationic surfactant in all the supporting electrolyte solutions studied is also confirmed by a decrease in the values of activation resistance R_a_ (determined by the electrochemical Faradaic impedance method) associated with the electroreduction reaction. The process of slowing down the Bi(III) ion electroreduction by the anionic surfactant is reflected by the increasing values of activation resistance in the surfactant solutions compared to the resistance determined for the Bi(III) ion electroreduction in the supporting electrolytes (Table 1 and Figure 6).

It should also be pointed out that there are differences in the values of activation resistance R_a_ with the change in the amount of methanol in the supporting electrolyte (Table 1), which indicates changes in the dynamics of the catalytic effect of CTAB and 1OSASS inhibition on the electrode process.

Literature data [2] display that the Zn(II) ion is selectively hydrated in mixed electrolyte solutions, which was evidenced by the obtained values of the relative Walden product of H_2_O + Et(OH) mixtures as a function of EtOH concentration and small values of Gibbs free energy of transition ∆G as a function of solution composition. In addition, we found that the cathodic transition coefficient α practically does not change with a change in the amount of ethanol in the supporting electrolyte. As a consequence, this suggests that the mechanism of the Zn(II) ions reduction electrode process in H_2_O + Et(OH) mixtures is the same as in aqueous solution [2].

The Bi(III) ion electroreduction mechanism on a mercury electrode is complex. Lovrič suggests a combination of potential–independent steps (partial dehydration of the reactant) with potential–dependent electron transfer steps and the following electrode mechanism [22]:

[Bi(H_2_O)_9_]^3+^ + e^−^ → [Bi(H_2_O)_4_]^2+^ + 5H_2_O

[Bi(H_2_O)_4_]^2+^ + e^−^ → [Bi(H_2_O)_2_]^+^ + 2H_2_O

[Bi(H_2_O)_2_]^+^ + e^−^ → Bi(Hg) + 2H_2_O

This reasoning has also been shown to be valid for the use of amalgam electrodes.

Suggestions toward similar observations of a multi-step electrode mechanism may also hold in the mixed electrolyte solution systems studied. An increase in the amount of methanol in the SE solution does not cause significant changes in the calculated values of the cathodic transition coefficients α (α_A_ = 0.35; α_B_ = 0.34; α_C_ = 0.33), which indicates the same electrode process mechanism in both aqueous and mixed water–organic solutions [2].

The “cap-pair rule” explains that an active complex formed on the electrode surface between Bi(III) ions and the organic substance is responsible for the effect of accelerating the Bi(III) ion electroreduction on mercury, but also on amalgam electrodes, including R-AgLAFE, by selected organic substances. It enhances electron transfer between the electrode and the depolarizer.

Small changes in the difference in the anodic and cathodic peak potentials ∆E along with the change in the rate of polarization (Table 2 and Table 3) in the studied systems indicate that the rate of the Bi(III) ion electroreduction is controlled by a chemical reaction.

This reaction is most likely the formation of Bi–surfactant active complexes on the electrode surface, which mediate electron transfer. The adsorption mentioned above of these compounds on the R-AgLAFE/supporting electrolyte interface, therefore, does not limit the electrode surface but shifts favorably the equilibrium of Bi(III) ion complexation that occurs in the adsorption layer.

The lack of significant changes in the values of the formal potential of Bi(III) ion electroreduction in all studied mixed supporting electrolytes with increasing surfactant concentration proves that no stable complexes of Bi(III) ions with CTAB or 1OSASS are formed in solutions (Table 4).

The differences in the kinetics of the Bi(III) ion electroreduction process associated with the presence of CTAB and 1OSASS are probably due to their structure and arrangement on the electrode. However, it seems more reasonable to conclude that the variety of active complexes mediating the electron transition, or their access to the electrode, play a key role [11,15].

As mentioned earlier, the organic component of mixed aqueous–organic supporting electrolytes (in this case, it is methanol), in a specific range of electrode potentials, modifies the electrode/solution interface, which is not without influence on the reaction kinetics. This can result in surface blocking and a subsequent possible decrease in the amount of surfactant–depolarizer active complexes in the adsorption layer. CTAB modifies the active surface of the electrode and, despite the greater adsorption than 1OSASS, catalyzes Bi(III) ion electroreduction. This may be due to the formation of structures at the electrode/solution interface, which by their structure and arrangement favorably affect an electron transfer.

In contrast, 1OSASS can form structures with different electrochemical activity and modify the electrode surface in a different way than CTAB. Thus, the active complexes that form, despite potentially accelerating the electrode process, cannot access the electrode surface. By adsorbing on the electrode, 1OSASS blocks access to it, and the additional presence of methanol in the supporting electrolyte solution reinforces this blockage. The measured true rate constants k_f_ (taking into account the influence of the double layer) of the Bi(III) ion electroreduction as a function of the electrode potential (the nonlinearity of this relationship), determined from impedance measurements [23,24,25,26] indicate that also in the presence of the tested surfactants in all the electrolyte solutions studied, the Bi(III) ion electroreduction process proceeds in a step-wise way. Moreover, the effect of surfactants on the passage of the first electron may be greater than on the passage of the remaining electrons. This is suggested by previous studies [27]. That states evidence that complexes of Bi(III) ions with surfactants can already be formed before the first electron transition, which is the slowest stage and determines the speed of the whole process. These active complexes also contribute to the passage of subsequent electrons. Nevertheless, it should be noted that the composition of active complexes after the passing of successive electrons changes, especially taking into account the variable composition of the supporting electrolyte.

The presence and increase in the amount of methanol in the supporting electrolyte in the absence of surfactants, resulting in changes in the values of the rate constants of the electrode process, is probably related to the decreasing hydration of the pre-electrode layer and weaker Bi(III) ion aqua-complex hydration. The selective hydration of metal ions in solutions containing methanol should also be mentioned [2,28].

A similar effect is also seen in the presence of surfactants. Thus, the presence of methanol does not adequately optimize the state of the pre-electrode layer for more efficient acceleration of the Bi(III) ion electroreduction by CTAB molecules. Hence, the catalytic effect of this surfactant declines its dynamics. The reason for the inhibition of the electrode reaction by the anionic surfactant related to the limited access of Bi—1OSASS active complexes to the electrode should also be related to the increase in the electrode overpotential, which takes place due to the presence of a mixed adsorption layer (due to the presence of MeOH in the supporting electrolyte solution) [28,29,30].

### 2.2. Kinetics Parameters

The determined kinetic parameters of the cathodic transition coefficient α and the standard rate constant k_s_ (with an accuracy of ±5%) based on CV curves quantitatively indicated the kinetics of the Bi(III) ion electroreduction process and in the presence of surfactants, as well as its changes due to the presence of methanol in the SE solution (Table 4).

Increasing the concentration of CTAB causes an increase in both α and k_s_ values, indicating an enhancement in the rate of the electrode process (Table 5). In contrast, in the presence of 1OSASS, the opposite situation is observed (Table 5). Both parameters decrease with the growth of the surfactant concentration in the supporting electrolyte solution, which clearly demonstrates changes in the direction of inhibition. The presence of methanol changes the dynamics of the catalytic action of CTAB and 1OSASS inhibition on the electrode process.

The literature reports that in mixed electrolyte solutions, the [Bi(H_2_O)_9_]^+3^ ion is specifically hydrated [31,32]. This suggests that dehydration of the Bi(III) ion plays a key role in accelerating the electroreduction process. The surfactant, by eliminating water molecules from the coordination sphere of the depolarizer, may facilitate this process. This fact indicates that this electroreduction reaction, both in the studied mixed electrolyte solutions and in the presence of chlorate(VII), proceeds through a complex of similar structure [27,28,29,30,31].

Readings in the literature strongly suggest the same structure of the active complex mediating electron transition in the electrode process in chlorate(VII) and mixed, water–organic electrolyte solutions, in which Bi(III) is selectively hydrated [28,29,30,31,32].

The magnitude of the catalytic effect of CTAB is related to the equilibrium reaction of the formation of active complexes before the passage of subsequent electrons. 1OSASS, as already mentioned, forming active complexes with Bi(III) ions that support the electroreduction process blocks access to the electrode surface. In addition, adsorbed methanol molecules strengthen this phenomenon. The adsorption layer is not unsealed, hence the inhibition, the magnitude of which should probably be related to the degree of electrode coverage.

## 3. Materials and Methods

### 3.1. Apparatus

An μAutolab FRA 2/GPES 4.9 potentiostat from Eco Chemie (Utrecht, The Netherlands) was used in the experiments, with a M165D programmable electrode stand (mtm-anko, Krakow, Poland) equipped with a three-electrode system. As a reference electrode, an Ag/AgCl/3M KCl electrode was used. A platinum wire served as the auxiliary electrode, while an innovative electrode made of cyclic renewable film of liquid silver amalgamate (R-AgLAFE) was used as the working electrode, with a surface area of 0.1741 cm^2^. This electrode is an effective alternative to the traditional mercury electrode (HMDE), having comparable performance and quality parameters, with a significant reduction in the consumption of harmful mercury [27,33].

Optimum experimental conditions were established for voltammetric or polarographic measurements: a scan rate of 2 mV∙s^−1^ was used for DC, while a scan rate of 5 to 1000 mV∙s^−1^ was used for cyclic voltammetry (CV), with a potential step of 2 mV, a pulse amplitude of 20 mV, and a frequency of 120 Hz for square wave voltammetry (SWV). Impedance experiments were carried out in the frequency range from 50 to 50,000 Hz using a 10 mV amplitude sinusoidal signal at an open circuit potential (OCP). To ensure repeatability and accuracy of the results, each measurement was carried out by performing at least three scans. 

### 3.2. Reagents and Solutions

The main source of the Bi(III) ions in the present study was bismuth(III) nitrate 5 hydrate (Bi(NO_3_)_3_ × 5H_2_O) (Sigma-Aldrich, St. Louis, MO, USA; CAS: 10035-06-0). The concentration of Bi(III) ions in the prepared solutions was always 1 × 10^−3^ mol∙dm^−3^. For the preparation of supporting electrolyte solutions, NaClO_4_ (Fluka, Buchs, Switzerland; CAS: 7601-89-0), HClO_4_ (Sigma-Aldrich; CAS: 7601-90-3) and ultrapure water, purified by the Millipore Milli-Q system (Millipore Sigma, Burlington, MA, USA), were used. The choice of chlorate(VII) as the primary electrolyte medium was justified by its minimal adsorption on the electrode surface and poor complex-forming properties [34,35]. Mixed aqueous–organic solutions of supporting electrolytes were prepared using different chlorate(VII)/methanol concentration ratios, with the chlorate(VII) concentration always being 1 mol-dm^−3^. The solutions were indicated as follows:

- Solution A: 1 mol∙dm^−3^ aqueous solution of chlorate(VII), without the addition of methanol (A—0%_(*v*/*v*)_ MeOH) (chlorate(VII):methanol ratio = 4:0).
- Solution B: 1 mol∙dm^−3^ mixed solution of chlorate(VII) in water with the addition of methanol, where the ratio of chlorate(VII):methanol = 3:1 (B—25%_(*v*/*v*)_ MeOH).
- Solution C: 1 mol∙dm^−3^ mixed solution of chlorate(VII) in water with the addition of methanol, where the ratio of chlorate(VII):methanol = 2:2 (B—50%_(*v*/*v*)_ MeOH).

To study the effect of ionic surfactants on Bi(III) ion electroreduction, the following substances were used: cationic surfactant hexadecyltrimethylammonium bromide (CTAB; Sigma-Aldrich; CAS: 2083-68-3) and anionic surfactant sodium 1-octane sulfonate (1OSASS; Fisher Scientific, Hampton, NH, USA; CAS: 5324-84-5). The concentrations of these compounds in solutions A, B, and C ranged from 5 × 10^−5^ mol∙dm^−3^ to 1 × 10^−3^ mol∙dm^−3^. All solutions were always prepared just before the measurements were taken and vented with high-purity nitrogen.

## 4. Conclusions

The presented comprehensive study of the Bi(III) ion electroreduction in aqueous and mixed aqueous–alcoholic chlorate(VII) solutions using an electrode of cyclic–renewable liquid silver amalgam (R-AgLAFE) clearly exposes that:

- The kinetics of the electrode process are affected by both the composition of the base electrolyte and the presence of ionic surfactants—hexadecyltrimethylammonium bromide and sodium 1-octane sulfonate.
- Adsorption of these surfactants on the surface of the R-AgLAFE electrode is determined by their ionic nature.
- Changes in the concentration of methanol and selected surfactants affect the rate of the Bi(III) ion electroreduction but do not change the mechanism of this process.
- CTAB accelerates the multi-step Bi(III) ion electroreduction process, while 1OSASS acts as an inhibitor.

Speaking about the mechanism of electroreduction of Bi(III) ions, its multi-stage nature was pointed out:

1. Formation of Active Complex I with the participation of adsorbed surfactant molecules (Surf) and partial dehydration of Bi(III) ions.
  Bi(H_2_O)_9_^3+^ + x(Surf)_ads._ → [Bi(H_2_O)_(9-a)_^3+^(Surf)_x_] + aH_2_O
2. The transition of the first electron.
  [Bi(H_2_O)_(9-a)_^3+^(Surf)_x_] + e^−^ [Bi(H_2_O)_(9-a)_^2+^(Surf)_x_]
3. Further dehydration with the formation of Active Complex II.
  [Bi(H_2_O)_(9-a)_^2+^(Surf)_x_] + y(Surf)_ads._ → [Bi(H_2_O)_(9-a-b)_^2+^(Surf)_x+y_] + bH_2_O
4. Second electron transition.
  [Bi(H_2_O)_(9-a-b)_^2+^(Surf)_x+y_] + e^−^ → [Bi(H_2_O)_(9-a-b)_^+^(Surf)_x+y_]
5. Further dehydration with the formation of Active Complex III.
  [Bi(H_2_O)_(9-a-b)_^+^(Surf)_x+y_] + z(Surf)_ads._ → [Bi(H_2_O)_(9-a-b-c)_^+^(Surf)_x+y+z_] + cH_2_O
6. Transition of the third electron and formation of an amalgam.
  [Bi^+^(Surf)_x+y+z_] + e^−^ → Bi(Hg) + (x + y + z)(Surf)_ads._

The presence of methanol modifies the dynamics of kinetic effects, highlighting the complexity of the influence of surfactants and electrolyte composition on the efficiency of the electrode process. Due to adsorption on the electrode surface, methanol competes for sites with Bi(III) ions and surfactant moieties, leading to a reduction in available active adsorption sites. This limits the ability to form stable Active Complexes I, II, and III, which translates into a reduction in the efficiency of electroreduction, especially in the initial stages of the mechanism. Therefore, methanol acts as an agent that slows down the entire process of Bi(III) ion electroreduction, although it does not change its multi-step nature.

The results of this work may have important implications for the development of modern electrode materials, corrosion inhibitors, or the optimization of existing technological processes.

## Figures and Tables

**Table 1:** Activation resistance (R_a_) values for the Bi(III) ion electroreduction in the presence of different concentrations of surfactants CTAB and 1OSASS.

C_CTAB_/mol∙dm^−3^	R_a_/Ω·cm^2^	C_1OSASS_/mol∙dm^−3^	R_a_/Ω·cm^2^
Solution A (1 mol∙dm^−3^ chlorate(VII) ions)			
0	121	0	121
5 × 10^−5^	1.56	5 × 10^−5^	123.03
1 × 10^−4^	1.08	1 × 10^−4^	128.67
1 × 10^−3^	0.39	1 × 10^−3^	132.45
**Solution B (1 mol∙dm^−3^ chlorate(VII) ions + 25%_(*v*/*v*)_ MeOH)**			
0	172.40	0	172.40
5 × 10^−5^	3.03	5 × 10^−5^	176.74
1 × 10^−4^	1.96	1 × 10^−4^	183.32
1 × 10^−3^	0.91	1 × 10^−3^	198.14
**Solution C (1 mol∙dm^−3^ chlorate(VII) ions+ 50%_(*v*/*v*)_ MeOH)**			
0	196.47	0	196.47
5 × 10^−5^	4.68	5 × 10^−5^	202.90
1 × 10^−4^	2.41	1 × 10^−4^	208.39
1 × 10^−3^	- *	1 × 10^−3^	211.43

* The substance did not dissolve.

**Table 2:** Impact of polarization rate on changes in ΔE of the 1 × 10^−3^ mol·dm^−3^ Bi(III) ion electroreduction of in aqueous–organic solutions of SEs and in the presence of selected concentrations of CTAB.

C_CTAB_/mol∙dm^−3^	∆E/V/V/mV·s^−1^							
	5	10	20	50	100	200	500	1000
Solution A (1 mol∙dm^−3^ chlorate(VII) ions)								
0	0.074	0.072	0.073	0.088	0.070	0.074	0.089	0.099
5 × 10^−5^	0.069	0.065	0.064	0.059	0.079	0.073	0.099	0.090
1 × 10^−4^	0.039	0.040	0.044	0.060	0.075	0.070	0.083	0.079
1 × 10^−3^	0.029	0.025	0.027	0.055	0.055	0.060	0.073	0.077
**Solution B (1 mol∙dm^−3^ chlorate(VII) + 25%_(*v*/*v*)_ MeOH)**								
0	0.084	0.089	0.090	0.132	0.171	0.181	0.230	0.259
5 × 10^−5^	0.078	0.076	0.078	0.088	0.078	0.083	0.101	0.140
1 × 10^−4^	0.043	0.044	0.045	0.090	0.070	0.079	0.093	0.139
1 × 10^−3^	0.033	0.032	0.032	0.095	0.095	0.089	0.094	0.130
**Solution C (1 mol∙dm^−3^ chlorate(VII)+ 50%_(*v*/*v*)_ MeOH)**								
0	0.088	0.090	0.098	0.151	0.180	0.191	0.240	0.264
5 × 10^−5^	0.079	0.078	0.079	0.093	0.120	0.130	0.191	0.201
1 × 10^−4^	0.051	0.053	0.052	0.090	0.110	0.120	0.180	0.190
1 × 10^−3^ *	-	-	-	-	-	-	-	-

* The substance did not dissolve.

**Table 3:** Impact of polarization rate on changes in ΔE of the 1 × 10^−3^ mol·dm^−3^ Bi(III) ion electroreduction of in aqueous–organic solutions of SEs and in the presence of selected concentrations of 1OSASS.

C_1OSASS_ [mol∙dm^−3^]	∆E/V/v/mV·s^−1^							
	5	10	20	50	100	200	500	1000
Solution A (1 mol∙dm^−3^ chlorate(VII) ions)								
0	0.074	0.072	0.073	0.088	0.070	0.074	0.089	0.099
5 × 10^−5^	0.083	0.083	0.081	0.100	0.122	0.156	0.180	0.208
1 × 10^−4^	0.085	0.087	0.090	0.112	0.127	0.158	0.183	0.220
1 × 10^−3^	0.089	0.090	0.093	0.122	0.132	0.162	0.187	0.213
**Solution B (1 mol∙dm^−3^ chlorate(VII) ions + 25%_(_***_v_*_/*v*_**_)_** **MeOH)**								
0	0.084	0.089	0.090	0.132	0.171	0.181	0.230	0.259
5 × 10^−5^	0.097	0.093	0.094	0.137	0.178	0.185	0.235	0.260
1 × 10^−4^	0.100	0.100	0.104	0.139	0.207	0.208	0.239	0.269
1 × 10^−3^	0.105	0.107	0.106	0.143	0.210	0.214	0.243	0.272
**Solution C (1 mol∙dm^−3^ chlorate(VII) ions + 50%_(_***_v_*_/*v*_**_)_** **MeOH)**								
0	0.088	0.090	0.098	0.151	0.180	0.191	0.240	0.264
5 × 10^−5^	0.100	0.107	0.104	0.154	0.179	0.196	0.239	0.265
1 × 10^−4^	0.109	0.103	0.107	0.159	0.210	0.217	0.247	0.273
1 × 10^−3^ *	0.122	0.123	0.121	0.163	0.215	0.219	0.257	0.277

* The substance did not dissolve.

**Table 4:** Values of formal potentials (E_f_^0^) and reversible half-wave potentials (E_1/2r_) of the 1 × 10^−3^ mol·dm^−3^ Bi(III) ion electroreduction in aqueous–organic solutions of supporting electrolytes and in the presence of selected concentrations of surfactants.

C_surf./_mol·dm^−3^/Surfactnt	E_f0_/V	E_1/2r_/V	C_surf./_mol·dm^−3^ /Surfactant	E_f0_/V	E_1/2r_/V
Solution A (1 mol∙dm^−3^ chlorate(VII) ions)					
0	0.016	0.010	0	0.016	0.0100
5 × 10^−5^ CTAB	0.013	0.014	5 × 10^−5^ 1OSASS	0.020	0.0035
1 × 10^−4^ CTAB	0.015	0.010	1 × 10^−4^ 1OSASS	0.016	0.0083
1 × 10^−3^ CTAB	0.016	0.011	1 × 10^−3^ 1OSASS	0.018	0.0081
**Solution B (1 mol∙dm^−3^ chlorate(VII) ions + 25%_(*v*/*v*)_ MeOH)**					
0	0.031	0.014	0	0.031	0.014
5 × 10^−5^ CTAB	0.016	0.013	5 × 10^−5^ 1OSASS	0.30	0.013
1 × 10^−4^ CTAB	0.025	0.020	1 × 10^−4^ 1OSASS	0.028	0.014
1 × 10^−3^ CTAB	0.025	0.021	1 × 10^−3^ 1OSASS	0.027	0.018
**Solution C (1 mol∙dm^−3^ chlorate(VII) ions + 50%_(*v*/*v*)_ MeOH)**					
0	0.037	0.230	0	0.037	0.230
5 × 10^−5^ CTAB	0.040	0.039	5 × 10^−5^ 1OSASS	0.044	0.042
1 × 10^−4^ CTAB	0.042	0.035	1 × 10^−4^ 1OSASS	0.042	0.038
1 × 10^−3^ CTAB *	-	-	1 × 10^−3^ 1OSASS	0.041	0.032

* The substance did not dissolve.

**Table 5:** Values of cathodic transition coefficient (α) and standard rate constants (k_s_) of the 1 × 10^−3^ mol·dm^−3^ Bi(III) ion electroreduction in mixed, aqueous–organic solutions of SEs and in the presence of selected concentrations of surfactants.

C_surf._/mol·dm^−3^/Surfactnt	α	10^4^ k_s_/cm·s^−1^	C_surf./_mol·dm^−3^/Surfactat	α	10^4^ k_s/_cm·s^−1^
Solution A (1 mol∙dm^−3^ chlorates(VII))					
0	0.33	1.30	0	0.33	1.30
5 × 10^−5^ CTAB	0.44	32.40	5 × 10^−5^ 1OSASS	0.025	1.00
1 × 10^−4^ CTAB	0.57	54.41	1 × 10^−4^ 1OSASS	0.023	0.95
1 × 10^−3^ CTAB	0.68	76.50	1 × 10^−3^ 1OSASS	0.020	0.84
**Solution B (1 mol∙dm^−3^ chlorate(VII) + 25%_(*v*/*v*)_ MeOH)**					
0	0.32	0.70	0	0.32	0.70
5 × 10^−5^ CTAB	0.41	7.07	5 × 10^−5^ 1OSASS	0.22	0.80
1 × 10^−4^ CTAB	0.55	34.70	1 × 10^−4^ 1OSASS	0.20	0.40
1 × 10^−3^ CTAB	0.60	49.10	1 × 10^−3^ 1OSASS	0.17	0.18
**Solution C (1 mol∙dm^−3^ chlorate(VII + 50%_(*v*/*v*)_ MeOH)**					
0	0.31	0.46	0	0.31	0.46
5 × 10^−5^ CTAB	0.380	4.06	5 × 10^−5^ 1OSASS	0.200	0.68
1 × 10^−4^ CTAB	0.490	12.88	1 × 10^−4^ 1OSASS	0.180	0.21
1 × 10^−3^ CTAB *	-	-	1 × 10^−3^ 1OSASS	0.160	0.12

* The substance did not dissolve.

## Data Availability

The original contributions presented in the study are included in the article, further inquiries can be directed to the corresponding author.
